# Targeting the Ubiquitin-Proteasome System in Limb-Girdle Muscular Dystrophy With CAPN3 Mutations

**DOI:** 10.3389/fcell.2022.822563

**Published:** 2022-03-02

**Authors:** Jaione Lasa-Elgarresta, Laura Mosqueira-Martín, Klaudia González-Imaz, Pablo Marco-Moreno, Gorka Gerenu, Kamel Mamchaoui, Vincent Mouly, Adolfo López de Munain, Ainara Vallejo-Illarramendi

**Affiliations:** ^1^ Group of Neuroscience, Departments of Pediatrics and Neuroscience, Faculty of Medicine and Nursing, Hospital Donostia, UPV/EHU, San Sebastian, Spain; ^2^ IIS Biodonostia, Neurosciences Area, Group of Neuromuscular Diseases, San Sebastian, Spain; ^3^ CIBERNED, Instituto de Salud Carlos III, Ministry of Economy and Competitiveness, Madrid, Spain; ^4^ Department of Physiology, Faculty of Medicine and Nursing, UPV/EHU, Leioa, Spain; ^5^ Sorbonne Université, Inserm, Institut de Myologie, Centre de Recherche en Myologie, Paris, France

**Keywords:** calpain 3 (CAPN3), calcium, muscular dystrophies, LGMD2A, SERCA1, SERCA2, bortezomib (BTZ)

## Abstract

LGMDR1 is caused by mutations in the *CAPN3* gene that encodes calpain 3 (CAPN3), a non-lysosomal cysteine protease necessary for proper muscle function. Our previous findings show that CAPN3 deficiency leads to reduced SERCA levels through increased protein degradation. This work investigates the potential contribution of the ubiquitin-proteasome pathway to increased SERCA degradation in LGMDR1. Consistent with our previous results, we observed that CAPN3-deficient human myotubes exhibit reduced SERCA protein levels and high cytosolic calcium concentration. Treatment with the proteasome inhibitor bortezomib (Velcade) increased SERCA2 protein levels and normalized intracellular calcium levels in CAPN3-deficient myotubes. Moreover, bortezomib was able to recover mutated CAPN3 protein in a patient carrying R289W and R546L missense mutations. We found that CAPN3 knockout mice (C3KO) presented SERCA deficits in skeletal muscle in the early stages of the disease, prior to the manifestation of muscle deficits. However, treatment with bortezomib (0.8 mg/kg every 72 h) for 3 weeks did not rescue SERCA levels. No change in muscle proteasome activity was observed in bortezomib-treated animals, suggesting that higher bortezomib doses are needed to rescue SERCA levels in this model. Overall, our results lay the foundation for exploring inhibition of the ubiquitin-proteasome as a new therapeutic target to treat LGMDR1 patients. Moreover, patients carrying missense mutations in CAPN3 and presumably other genes may benefit from proteasome inhibition by rescuing mutant protein levels. Further studies in suitable models will be necessary to demonstrate the therapeutic efficacy of proteasome inhibition for different missense mutations.

## Introduction

Limb-girdle muscular dystrophy recessive 1 (LGMDR1), or calpainopathy (previously known as LGMD2A), is the most common form of limb-girdle dystrophies. It is caused by mutations in the CAPN3 gene, which encodes the proteolytic enzyme calpain 3 (CAPN3), a non-lysosomal cysteine protease (Richard et al., 1995). This disease is characterized by progressive degeneration of scapular and pelvic girdles, together with proximal lower limb muscles (Richard et al., 2016). The pathological mechanism underlying this disease remains unclear and, currently, there is no effective therapy for these patients. To date, most therapeutic strategies for LGMDR1 are focusing on correcting the primary genetic defect through gene or cell therapies (Roudaut et al., 2013; Straub and Bertoli, 2015; Lasa-Elgarresta et al., 2019; Selvaraj et al., 2019; Sahenk et al., 2021). However, there is a need for developing novel pharmacological therapies directed towards alternative targets of the disease.

An increasing number of studies point to the dysregulation of Ca^2+^ homeostasis as a pathophysiological mechanism involved in LGMDR1. The absence of functional CAPN3 in *CAPN3* knockout mouse has been linked to a reduced expression of the ryanodine receptor type 1 (RyR1) and -reduced Ca^2+^ release from the sarcoplasmic reticulum (SR) (Kramerova et al., 2008; Dayanithi et al., 2009). Moreover, reduced RyR1 expression and CaMKII signaling in muscles from LGMDR1 patients and C3KO mice have been reported (Kramerova et al., 2012). On the other hand, *CAPN3* knockout myotubes show decreased SR Ca^2+^ levels and reduced response to specific sarco/endoplasmic reticulum calcium ATPase (SERCA) inhibitors (Dayanithi et al., 2009). In this line, our group has previously demonstrated decreased SERCA protein levels and impaired SERCA function in mouse and human CAPN3 deficient myotubes (Toral-Ojeda et al., 2016; 2018), while SERCA mRNA levels (*ATP2A1/ATP2A2*) remained unaffected. Likewise, we found reduced SERCA protein levels in muscle samples from LGMDR1 patients. Overall, these results suggest that CAPN3 is necessary to stabilize SERCA proteins and prevent their degradation. Of note, we found that CAPN3 deficiency caused an increase in resting intracellular [Ca^2+^] in human LHCN-M2 myotubes but not in mouse C2C12 myotubes (Toral-Ojeda et al., 2016), which is probably due to the higher Ca^2+^ buffering capacity of mouse muscle fibers (Toral-Ojeda et al., 2018).

The ubiquitin-proteasome system (UPS) and the autophagy-lysosome pathway are key cell proteolytic processes that control protein turnover in the muscle (Bonaldo and Sandri 2013). Upregulation of UPS has been previously reported in muscular dystrophies, and pharmacological inhibition of this pathway has been found to prevent the removal of mutated proteins, promote functional recovery, and improve the dystrophic phenotype associated with these disorders (Nastasi et al., 2004; Briguet et al., 2008; Gastaldello et al., 2008; Carmignac et al., 2011; Fanin, 2014; Körner et al., 2014; Rajakumar et al., 2014). In LGMDR1, UPS-mediated protein degradation seems to be the main pathway that leads to muscle atrophy since overexpression of UPS-related genes has been found to correlate with the severity of atrophy in muscle biopsies of LGMDR1 patients (Rajakumar et al., 2013; M. ; Fanin et al., 2013). Regarding SERCA1 and SERCA2 proteins, we have previously shown increased ubiquitination levels in CAPN3-deficient myotubes, suggesting that UPS inhibition could restore SERCA protein expression in LGMDR1 (Toral-Ojeda et al., 2016). Thus, in this work, we aimed to investigate the contribution of the UPS in LGMDR1 pathology and to evaluate whether inhibition of the UPS could rescue SERCA proteins in cellular and animal models of LGMDR1. To this end, we tested the effect of Bortezomib (BTZ), a UPS inhibitor that has been granted FDA approval for the treatment of multiple myeloma (Kane et al., 2003). In addition, BTZ has been previously tested in several models of muscular dystrophy with reported therapeutic potential (Araújo et al., 2009; Gazzerro et al., 2010; Carmignac et al., 2011; Körner et al., 2014).

## Materials and Methods

### Cell Cultures

Immortalized human myoblasts 8,220 (Echigoya et al., 2015; Echigoya et al., 2017), KM900, and 918 were kindly provided by Dr. Vincent Mouly (Myology Institute Paris, France). These cells were grown and differentiated as previously described (Toral-Ojeda et al., 2018), only without addition of an ECM overlay. KM900 myoblasts (LG1) are homozygous for G567W mutation (c.1699G>T, exon 13), while 918 myoblasts (LG2) present two mutations R289W (c.865C>T, exon 6) and R546L (c.1637G>T, exon 13). 8,220 control line was transduced with lentiviral particles containing shRNAs to knockdown *CAPN3* gene expression. Lentiviral particles were generated by the Viral Vector Unit (ViVU) at CNIC (Madrid), from plasmid TRCN0000003494 carrying a human-specific shRNA for *CAPN3*, and plasmid SHC002 carrying a non-mammalian shRNA control (MISSION^®^ pLKO.1-puro, Sigma-Aldrich). Myoblasts were transduced with lentivirus at MOI 5 with 4 μg/ml polybrene for 24 h. Afterwards, cells were differentiated to myotubes as described above. After 4 days in differentiation medium, myotubes were treated for 24 h with 5 nM BTZ (Selleckchem).

### Mouse Lines

The Calpain3 knockout mouse model (C3KO) was kindly donated by Dr. Spencer (UCLA) in a C57BL/6 genetic background (Kramerova et al., 2004). Mice were backcrossed with the C57BL/6 mice to homogenize genetic background between wild-types (controls) and C3KO mice. For genotyping, DNA was extracted from mouse tails using a rapid alkaline lysis protocol, as previously described (Lasa-Fernandez et al., 2020). Genotyping for CAPN3 was performed by using the following primers: LTR2, 5′-AAA​TGG​CGT​TAC​TTA​AGC​TAG​CTT​GC-3′; C3 forward, 5′-GAA​AGG​GAC​AGG​AGA​AAT​GGA​G-3′; C3 reverse, 5′-CCT​GAA​ACT​TCA​AGC​CTC​TGT​TC-3′. PCR was run according to the following conditions: an initial denaturation step at 95°C for 2 min followed by 30 cycles of 95°C for 20 s, 60°C for 20 s and 72°C for 1 min. Treatment was administered by intravenous tail injection every 72 h for 3 weeks under isoflurane anesthesia. C3KO mice were treated with 0.8 mg/kg BTZ (Velcade) diluted in saline (*n* = 8) or saline (*n* = 9). One C3KO mice died after the first injection (BTZ group), and thus, it was excluded from the analysis. All procedures were conducted in accordance with protocols approved by the Ethical Board Committee of Animal Care at Biodonostia Institute (CEEA 17_015).

### Western Blotting

Proteins were extracted from muscle tissues and cell cultures with 125 mM Tris/HCl, 10% glycerol, 1% SDS, 4M Urea, and 5% β-mercaptoethanol, pH 6.8. Protein extracts (7–14 µg/well) were resolved in precast 4–20% gradient SDS-PAGE gels (Mini-Protean, Bio-Rad). Proteins were transferred onto low fluorescence PVDF membranes, blocked with TBS-tween and 5% skim milk, and incubated with primary antibodies overnight at 4°C. Afterwards, membranes were incubated with fluorescent (Alexa Fluor 488, 647, and 800) or horseradish peroxidase-conjugated secondary antibodies. Images were acquired with an iBright FL1500 Imaging System apparatus (Thermo scientific), and Image Studio Lite 4.0 software was used for signal quantification. Specific signals were normalized with myosin heavy chain (MyHC) or total protein (No Stain Protein Labelling Reagent, Thermo Scientific). Antibodies were obtained from the following sources: Calpain 3 12A2 monoclonal antibody (mAb) (MONX10794, Monosan, 1:100 dilution); DHPRɑ2 mAb (ab2864, Abcam, 1:1000 dilution); GAPDH mAb (MAB374, Millipore, 1:5000 dilution), MyHC mAb (A4.1025, DSHB, 1:5000 dilution); P-CaMKII polyclonal antibody (pAb) (PA537833, Invitrogen, 1:500 dilution); RyR1 mAb (MA3-925, Thermo, 1:1000 dilution); SERCA1 mAb (MA3-912, Thermo, 1:1000 dilution); SERCA2 mAb (sc-376235, Santa Cruz, 1:250 dilution); SR-actin pAb (ab97378, Abcam, 1:5000 dilution), β-CaMKII mAb (13-9800, Invitrogen, 1:250 dilution).

### Real-Time Quantitative PCR (qPCR)

Total RNA from cell pellets was extracted with the miRNeasy Mini kit plus DNaseI (Qiagen), following the manufacturer’s instructions. cDNA was synthesized using SuperScript Vilo cDNA Synthesis Kit (Thermo Fisher). qPCR was performed and analyzed with the CFX384 system (Bio-Rad), using Power SYBR^®^ Green PCR Master Mix (Thermo Fisher). Primers for the genes of study were designed using Primer Express software (Thermo Fisher). Primer specificity was verified with the *in silico* PCR tool from The University of Santa Cruz (California), and primer dimers were determined with the Multiple Primer Analyzer tool from Thermo Fisher. Technical triplicate measurements were performed in at least three different cultures, and the results were normalized to a normalization factor based on the geometric mean of the four most stable reference genes: *CK*, *DHPRα1*, *DYST*, and *HPRT1* (Toral-Ojeda et al., 2016). Primer sequences used in this study are shown in Table 1.

**TABLE 1:** Sequence of primers used for real-time qPCR analysis.

Gene	Forward primer	Reverse primer
*ATP2A1*	TAC​GAT​GAG​ATC​ACA​GCC​ATG​AC	ATC​CCA​TGG​CAA​TGC​CAA​T
*ATP2A2*	AAA​GCT​AAA​GAC​ATA​GTT​CCT​GGT​GAT	AGC​AGG​ACT​TTG​TCA​CCA​ACA
*CAPN3*	CTG​TTC​AAA​GGT​GAG​AAG​GTG​AAG	AGC​TCC​AGT​CCT​TCC​AAC​CAT
*RyR1*	CCT​CAT​CAA​CTA​TGT​CAC​CAG​CAT	CCA​CCA​TCA​CCT​CAA​AGT​ACC​ATT
*CK*	GAA​GCT​CTC​TGT​GGA​AGC​TCT​CA	CCT​TCT​CCG​TCA​TGC​TCT​TCA
*DHPRα1*	GCC​ATC​TCC​GTG​GTG​AAG​AT	CAC​TGC​ACC​ACG​TGC​TTC​A
*DYST*	ACA​GGG​CAA​AAA​CTG​CCA​AA	CGC​AGT​GCC​TTG​TTG​ACA​TT
*HPRT1*	CAT​GGA​CTA​ATT​ATG​GAC​AGG​ACT​GA	TGA​GCA​CAC​AGA​GGG​CTA​CAA

### Calcium Imaging

Cytosolic calcium imaging was analyzed in human myotubes using the ratiometric calcium dye Fura-2AM as described previously (Toral-Ojeda et al., 2016). Briefly, cells were loaded with 4 µM Fura-2AM in the presence of 0.02% pluronic acid for 30 min at 37°C in culture medium. Cells were washed with Ringer solution (125 mM NaCl, 5 mM KCl, 1.2 mM MgSO4, 6 mM glucose, 2 mM CaCl2 and 25 mM HEPES, pH 7.4) and after 15 min, images were acquired with an ECLIPSE Ti/L100 microscope (Nikon) equipped with a ×20 S-Fluor objective, a lambda-DG4 illumination system, and an Orca-Flash 2.8 camera (Hamamatsu). NisElements-AR software was used for data analysis. Intracellular calcium concentration was estimated by the ratio of Fura-2AM fluorescence intensities at excitation wavelength 340 and 380 nm. To convert ratio values to concentration values the following equation was used: [Ca^2+^]_i_ = βK_D_(R-R_min_)/(R_max_-R), where K_D_ is the apparent dissociation constant of Fura-2AM (224 nM).

### Serum Creatine Kinase

Blood was extracted by intracardiac puncture and collected into BD Microtainer^®^ SST tubes. Samples were centrifuged at 6,000 g for 10 min at 4°C and serum was kept at −80°C until analysis. Creatine kinase (CK) determination was performed by the Biochemistry Service at Donostia University Hospital following a standardized photometric technique. Hemolyzed samples were not included in the analysis.

### Grip Strength Test

Forelimb grip strength was performed using a grip strength meter (Bioseb) following the standard procedure (Luca, 2008). Briefly, mice were lifted by the tail and left to grasp the grid with the forelimbs. Five consecutive measurements were done with a resting period of 1 min between them, and the three highest measurements were considered for data analysis. Data were normalized to the body weight.

### Fatigue Resistance

Mice were subjected to a running exhaustion experiment, based on a previously described protocol (Kramerova et al., 2016). First, mice were acclimated for 2 days to the treadmill (Columbus Instruments) with a 20 min run per day at 10 m/min. On the third day, mice run at 16 cm/s for the first 10 min (0–10 min), at 20 cm/s during the next 20 min (10–30 min), and at 24 cm/s during the next 30 min (30–60 min). Afterwards, the speed was increased by 4 cm/s every 5 min until the mouse was unable to continue running. A mouse was considered exhausted when it was not able to continue running on the treadmill for 1 min, and when during that time it received three or more electrical shocks of 0.4 mA (Capogrosso et al., 2018).

### Ubiquitin Proteasome Activity

Ubiquitin proteasome activity was measured in the soleus muscles of control and C3KO mice treated or not with BTZ, by using a previously described method based on the cleavage of a specific fluorogenic substrate (Gazzerro et al., 2010). Briefly, muscle samples were homogenized in 50 mM HEPES, 100 mM NaCl, pH 8.0 and centrifuged. The protein concentration of supernatants was determined using the Bradford Protein Assay (Bio-Rad). 12.5 µg protein were incubated for 60 min at 37°C in the presence of a specific fluorogenic substrate (LLVY-R110, Sigma-Aldrich). The fluorescent signal was read with a microplate reader (Glomax Discover, Promega) at 525 nm. Homogenization buffer was used as a negative control and 1 µM BTZ was added to the protein sample as a positive control. Background signal from the negative control was subtracted from sample signals and data was represented as percentage over control.

### Data Analysis and Statistical Procedures

GraphPad Prism version 6.04 was used to perform statistical analyses. Data distribution was evaluated with Shapiro-Wilk and D’Agostino & Pearson omnibus normality tests. Data having a Gaussian distribution were analyzed using unpaired, paired or ratio Student’s t-test. When comparing more than two groups, One-Way ANOVA followed by Dunnett or Tukey post-hoc test was used. When analyzing two factors, Two-Way ANOVA followed by Tukey post-hoc test was applied. When data did not follow a normal distribution, Mann-Whitney test or Kruskal Wallis test followed by Dunn’s post-hoc were used. For all statistical analyses, the adjusted *p*-values of less than 0.05 were considered statistically significant.

## Results

### Analysis of BTZ on Ca^2+^-Handling Proteins in CAPN3 Silenced Myotubes

**FIGURE 1:**
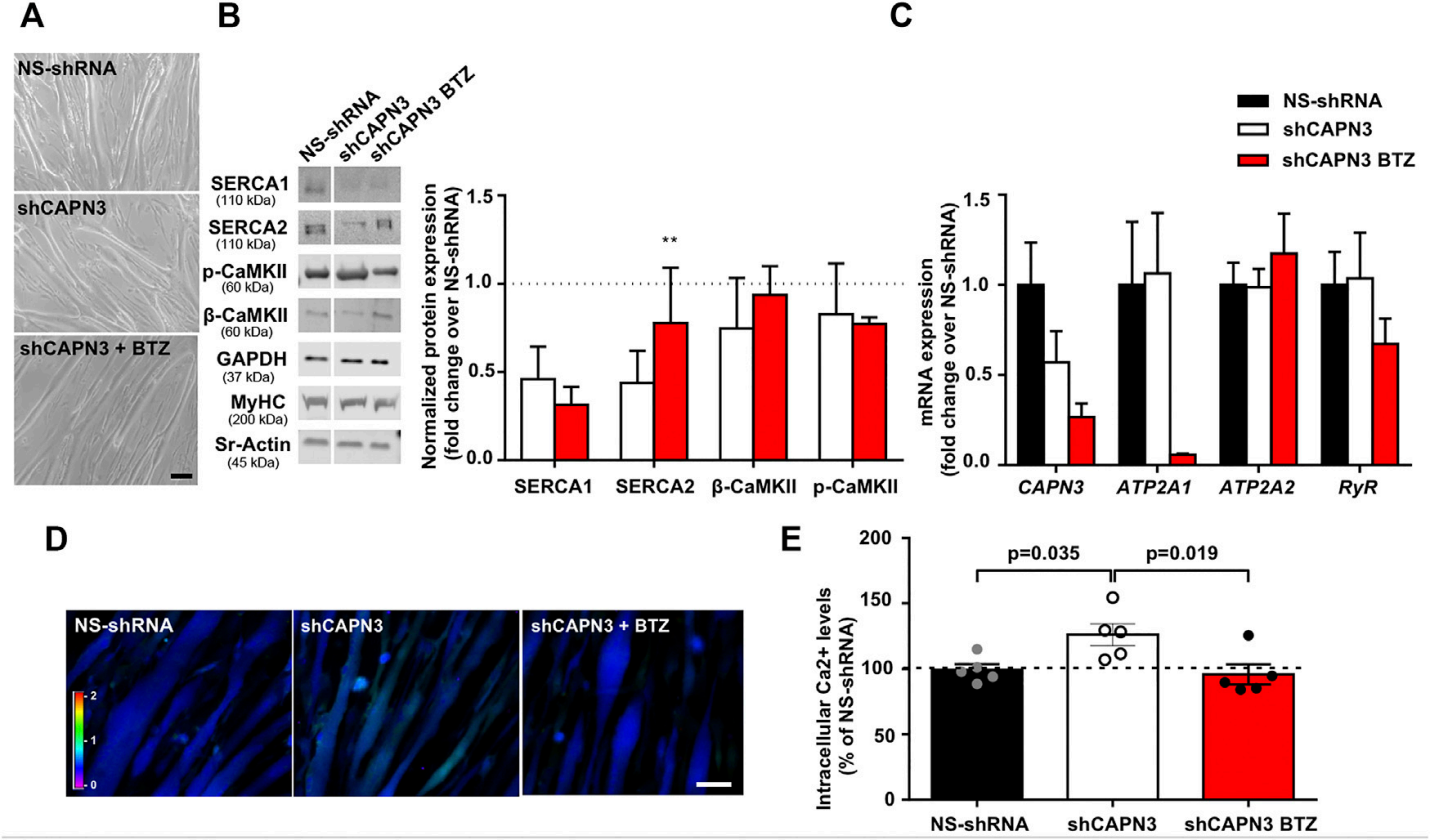
Effect of UPS inhibition in 8220 CAPN3-deficient human myotubes. **(A)** Representative bright-field images of human control (NS-shRNA) and CAPN3 knockdown (shCAPN3) myotubes after 5 days in differentiation. Treatment with 5 nM BTZ was performed for 24 h where indicated. Scale bar: 50 μm. **(B)** Western blot analysis of Ca^2+^-handling proteins in control and CAPN3-deficient cells treated or not with BTZ. White line depicts non-continuous lanes within the same blot. Protein signals are normalized to MyHC and expressed as fold change over each control (NS-shRNA), which is represented as a discontinuous line in the bar chart. Data are expressed as mean fold-change ± SEM of *n* = 3 independent experiments; **p < 0.01 vs. non-treated shCAPN3 (ratio paired *t*-test). **(C)** Analysis of mRNA expression in CAPN3-deficient myotubes with or without BTZ treatment. Data expressed as mean fold-change ± SEM over NS-shRNA. *n* = 3 independent experiments (one-way ANOVA post hoc Tukey’s multiple comparisons test). **(D)** Representative pseudocolored images of human myotubes (NS-shRNA, shCAPN3, and shCAPN3 treated with BTZ) loaded with Fura-2AM. Scale bar: 25 μm. **(E)** Bar chart shows the resting intracellular calcium levels of human myotubes. Data expressed as mean % over NS-shRNA ± SEM based on calcium concentration levels. Dots represent individual experiments (*n* = 5) with a total of 120–160 myotubes analyzed per group (one-way ANOVA post hoc Dunnett’s multiple comparisons test).

In a previous study, we reported reduced SERCA protein levels together with diminished SERCA ATPase activity in human (LHCN-M2) and mouse (C2C12) myotubes knockdown for CAPN3 (Toral-Ojeda et al., 2016). In this study, we used a different human immortalized myogenic line (8,820), which is more efficient than LHCN-M2 in generating highly mature myotubes. Indeed, in our hands 8,220 myoblasts were able to generate contractile myotubes after 5–7 days in differentiation medium with no need of an extracellular matrix overlay, in contrast to LHCN-M2 myoblasts (Toral-Ojeda et al., 2018). Similar to our previous work, CAPN3 expression was silenced in 8,220 myotubes with lentiviral particles carrying specific shRNA for CAPN3 (Toral-Ojeda et al., 2016; Toral-Ojeda et al., 2018). To analyze the role of UPS in LGMDR1, we treated 8220 CAPN3-deficient (shCAPN3) myotubes with 5 nM BTZ for 24 h. After treatment, no obvious morphological differences were found among the different samples (Figure 1A). In agreement with our previous findings, shCAPN3 myotubes showed a decrease of both SERCA1 and SERCA2 of around 50% compared to control myotubes (NS-shRNA, Figure 1B). Interestingly, in CAPN3-deficient myotubes, treatment with BTZ significantly increased SERCA2 protein levels by 34% compared to non-treated cells (*p* < 0.01, *n* = 3), whereas no significant effect on SERCA1 protein was observed. We also analyzed CaMK signaling pathway, which regulates contraction-induced Ca^2+^-handling and mitochondria biogenesis. In this case, we found no differences in the expression of β-CaMKII or its phosphorylated form in CAPN3-deficient myotubes, nor an alteration of the level of these proteins with BTZ treatment. At the mRNA level, we found that CAPN3 deficiency does not affect *ATP2A1* and *ATP2A2* expression, codifying for SERCA1 and SERCA2, respectively. This agrees with our previous results (Toral-Ojeda et al., 2016) and indicates that SERCA protein levels are reduced in CAPN3 knockdown myotubes due to an abnormal protein degradation. Unexpectedly, we found that BTZ treatment drastically downregulates *ATP2A1* expression by more than 90% (Figure 1C). This effect does not seem to be reflected at the protein level, but that might be due to the relatively short BTZ exposure (24 h).

Next, we performed calcium imaging to explore whether UPS inhibition affects intracellular calcium levels and SERCA function (Figures 1D,E). Indeed, we observed that BTZ treatment results in a significant rescue of intracellular calcium levels, which are increased in CAPN3-silenced myotubes relative to control myotubes (95.92 ± 7.66% shCAPN3 BTZ vs. 126.2 ± 8.34% shCAPN3, *p* = 0.019). This effect may be explained by the increase in SERCA2 observed at the protein level. These results suggest that BTZ predominantly rescues the loss of SERCA2 but not SERCA1 in the absence of CAPN3. Furthermore, in the CAPN3 knockdown model, SERCA2 seems to have a relevant effect on basal calcium levels.

### Analysis of BTZ on Ca^2+^-Handling Proteins in Immortalized Human Myotubes From LGDMR1 Patients

**FIGURE 2:**
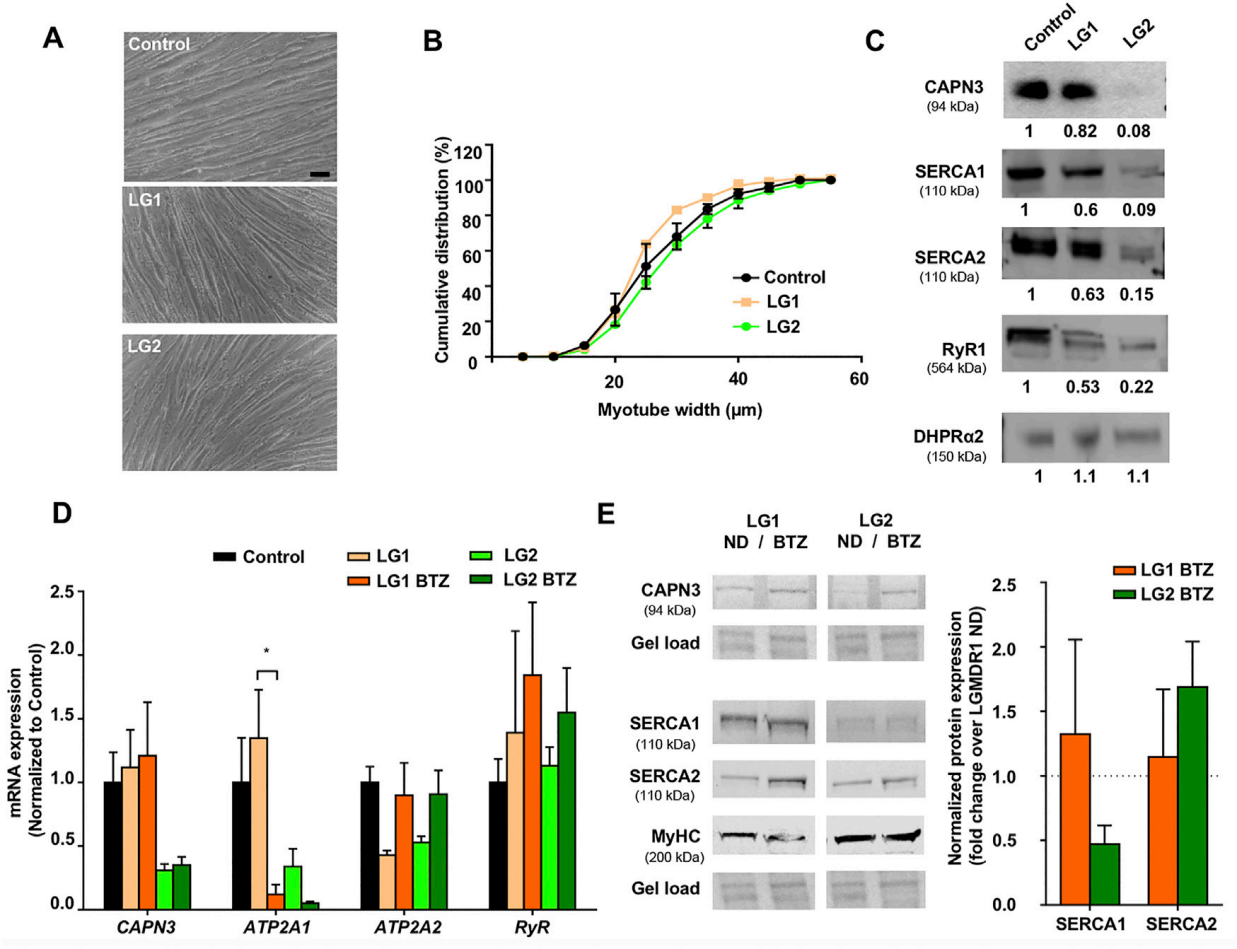
Effect of UPS inhibition in myotubes from LGMDR1 patients. **(A)** Representative bright-field images showing immortalized human myotubes from a control and two LGMDR1 patients (LG1 and LG2) after 5 days in differentiation. Scale bar: 50 μm. **(B)** Cumulative distribution of myotube width from control, LG1 and LG2 myotubes. 90–170 myotubes analyzed from *n* = 3 independent experiments. Data are expressed as mean % ± SEM (two-way ANOVA test). **(C)** Western blot analysis of CAPN3, SERCA1, SERCA2, RyR1, and DHPRɑ2. Quantification of protein signals is shown below each blot, represented as fold change over control. **(D)** qPCR expression analysis of Ca^2+^-handling proteins in myotubes from control and LGMDR1 patients, treated or not with BTZ. Data are expressed as mean ± SEM. *n* = 3 independent experiments. *p < 0.05, One-way ANOVA post hoc Tukey’s multiple comparisons test. **(E)** Representative western blot signals from LG1 and LG2 myotubes treated with 5 nM BTZ for 24 h after 5 days of differentiation and their quantification. Protein signals are normalized to total protein (gel load) and represented by fold change over non-treated LGMDR1 myotubes. Non-treated LGMDR1 SERCA expression levels are shown as a discontinuous line.

We next wanted to characterize the expression of Ca^2+^ handling proteins in myotubes from LGMDR1 patients. To this end, we used immortalized myogenic lines KM900 (LG1) and 918 (LG2) that carried several mutations in the *CAPN3* gene and 8,220 myotubes were used as healthy control for comparison. No obvious differences in myoblast growth and differentiation were observed among the different lines. LG1 myotubes did show a slightly thinner morphology (Figure 2A), however, cumulative distribution analysis of myotube width revealed no significant differences between control and LG myotubes when Two-way ANOVA statistical analysis was applied (Figure 2B). We then analyzed the expression of Ca^2+^-handling proteins in these samples, and observed that LG2 myotubes exhibited a massive loss of CAPN3 expression, whereas LG1 myotubes apparently had normal levels (Figure 2C). In addition, both LG1 and LG2 patient samples displayed decreased levels of SERCA1/2 and RyR1 proteins compared to control, while there appeared to be no change in DHPR protein levels among the three samples. This is in line with previous studies reporting reduced RyR1 protein levels in C3KO mouse muscle (Kramerova et al., 2008), LGMDR1 muscle biopsies (Kramerova et al., 2012), and *CAPN3* knockdown myotubes (Toral-Ojeda et al., 2016). Of note, LG2 myotubes displayed the lowest protein levels of CAPN3, SERCA1, SERCA2 and RyR1, while normal DHPR protein levels were preserved. At the mRNA level, LG2 myotubes also presented lower levels of *CAPN3*, *ATP2A1*, *ATP2A2* compared to control myotubes, whereas *RyR1* expression levels appeared unchanged (Figure 2D). After treatment with BTZ, we observed a significant downregulation of *ATP2A1* in LG1 myotubes (*p < 0.05, One-way ANOVA, Tukey’s post hoc test), and a similar trend was also observed in LG2 myotubes. This effect is analogous to that observed in CAPN3 knockdown myotubes treated with BTZ. Intriguingly, *ATP2A2* appeared to be upregulated with BTZ treatment in both LG1 and LG2 myotubes, although this effect did not reach statistical significance. At the protein level, SERCA2 appeared to be increased although not significantly in LG2 myotubes after BTZ treatment, while SERCA1 levels appeared to be reduced. These results closely resemble those of CAPN3 knockdown myotubes (Figure 1). In contrast, SERCA levels seemed unaffected by BTZ treatment in LG1 myotubes. Most surprising was the effect of BTZ observed on CAPN3 levels. While at the mRNA level BTZ did not alter *CAPN3* expression of LGMDR1 myotubes, we found that at the protein level BTZ treatment rescued the expression of CAPN3 in LG2 dystrophic myotubes (Figure 2E).

### C3KO Mouse Phenotype and SERCA Protein Expression at Preclinical Stage

Several Capn3-deficient mouse models recapitulate to some extent the pathophysiological features of LGMDR1 muscular dystrophy. Among them, we selected the C3KO mouse line generated by Dr. Spencer’s group (UCLA, United States), since it is one of the most thoroughly characterized models and Ca^2+^ dysregulation has been previously reported in this model (Kramerova et al., 2008). Our aim was to analyze SERCA protein expression in the early stages of the disease, in order to determine whether SERCA might be involved in the etiopathology of LGMDR1.

**FIGURE 3:**
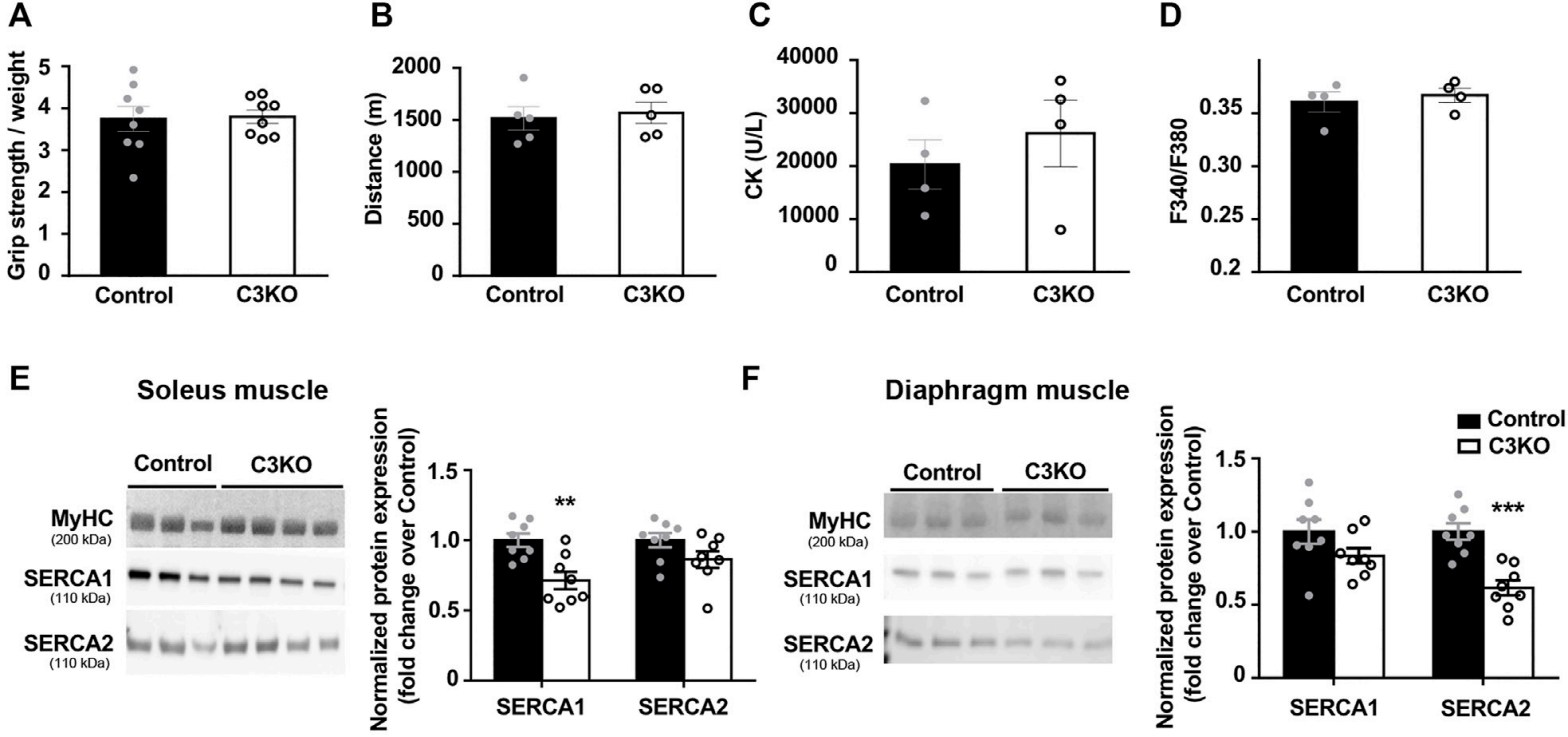
Characterization of early features in preclinical C3KO mice. **(A)** Grip strength normalized to body weight. Data expressed as mean ± SEM. *n* = 8 2-month-old male mice for each genotype (Mann-Whitney test). **(B)** Distance until exhaustion during forced exercise. Data expressed as mean ± SEM, *n* = 5 mice per group (Mann-Whitney test). **(C)** Serum creatine kinase (CK) levels. Data are expressed as mean ± SEM. *n*= 4 mice per group (Mann-Whitney test). **(D)** Resting intracellular calcium levels of fibers isolated from *flexor digitorum brevis* muscles. Data expressed as mean ± SEM. 115-155 fibers analyzed from *n* = 4 mice per group (Mann-Whitney test). **(E,F)** Western blot analyses of SERCA proteins normalized to MyHC in soleus **(E)** and diaphragm **(F)** muscles. Data are expressed as mean fold change ± SEM of *n* = 8 mice per genotype.**p < 0.01, ***p < 0.001 vs. control (Mann-Whitney test). Dots in the bar charts represent data from one mouse.

First, we characterized the phenotype of 2-month-old C3KO mice using standard techniques for this purpose. In our case, we observed no significant changes in grip strength (Figure 3A) or fatigue endurance (Figure 3B) of C3KO dystrophic mice compared to controls. We also analyzed serum CK levels, since hyper-CKemia has been described in LGMDR1 patients at early stages of the disease (Urtasun et al., 1998; Fanin and Angelini, 2015). However, no significant differences were seen in serum CK levels between C3KO and control mice (Figure 3C). Lastly, we analyzed basal intracellular Ca^2+^ levels in *flexor digitorum brevis* (FDB) muscle fibers isolated from young mice. In this analysis, we observed no significant differences between isolated fibers from WT and C3KO mice (Figure 3D). For the analysis of SERCA protein levels, we selected soleus and diaphragm muscles, since they are reportedly the most affected muscles in the C3KO model (Kramerova et al., 2004). Our results reveal that there is an overall decrease of SERCA proteins in C3KO mouse muscles at early stages of the disease. Specifically, in the soleus muscle we observed that SERCA1 expression in C3KO mice is significantly lower compared to WT mice (controls), decreasing by 28.7% (0.713 ± 0.062 in C3KO vs. 1 ± 0.047 in control; **p = 0.005). As for SERCA2 levels, they showed a tendency to decrease (13.71%), but did not reach statistical significance (Figure 3E). In contrast, in the diaphragm, we found that the levels of SERCA2 are significantly reduced, by 38.5% for SERCA2 (0.61 ± 0.052 in C3KO vs. 1.0 ± 0.056 in control; ***p = 0.0006). As for SERCA1 levels, they also showed a tendency to decrease (16.57%) but it did not reach statistical significance (Figure 3F). These results indicate that C3KO mice display SERCA deficits in skeletal muscle early in the disease, even before the manifestation of overt muscle deficits.

### SERCA Protein Expression in Adult C3KO Mice Upon BTZ Treatment

**FIGURE 4:**
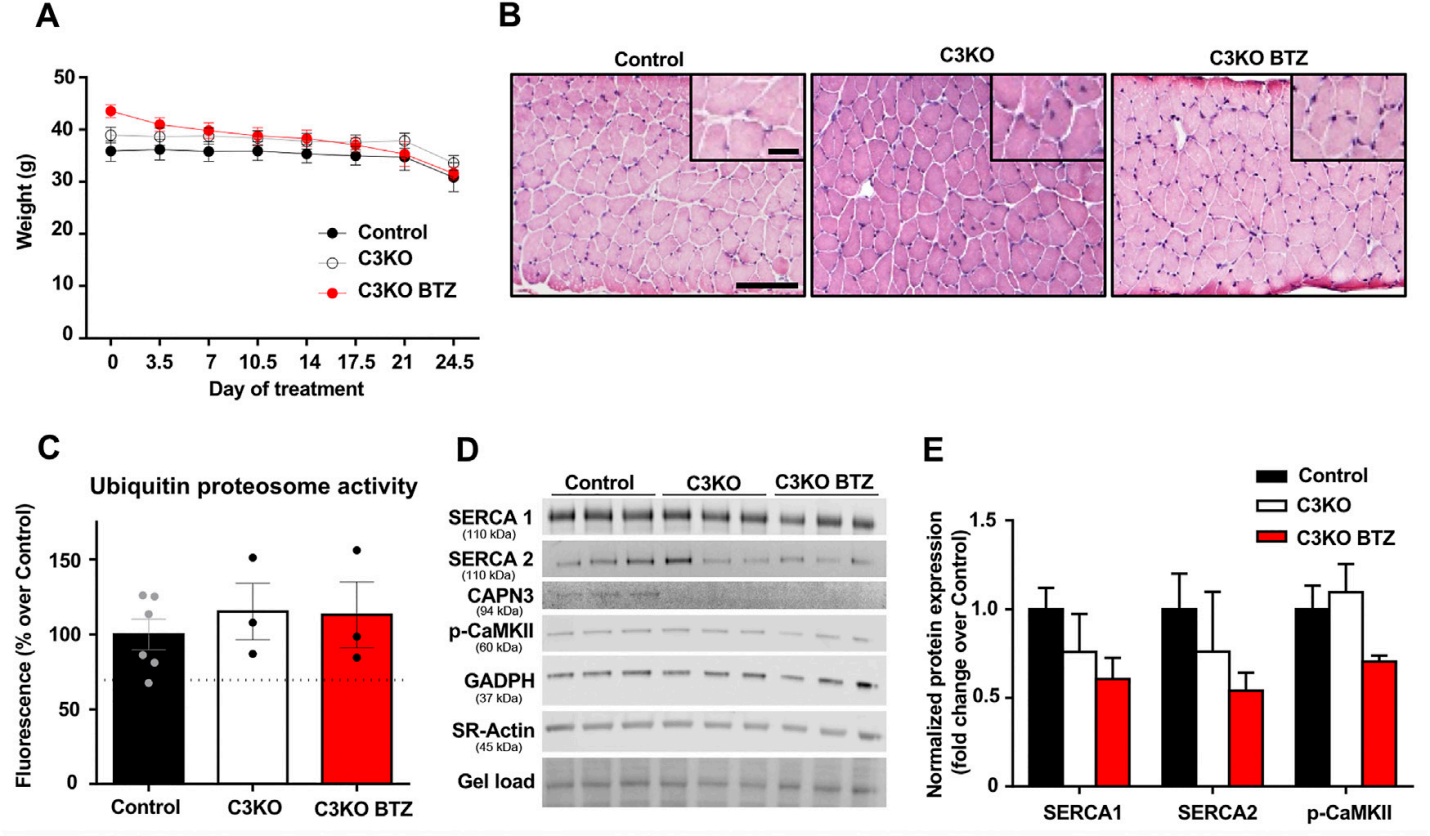
Effect of BTZ on C3KO mice. **(A)** Body weight evolution during BTZ treatment of 9-month-old mice. Data are presented as mean ± SEM. *n* = 10 non-treated wild-type mice (Control), *n* = 9 vehicle-treated C3KO mice (C3KO), and *n* = 7 BTZ-treated (0.8 mg/kg) C3KO mice (C3KO BTZ). (Two-way ANOVA post hoc Tukey’s multiple comparisons test. **(B)** Hematoxylin and eosin staining images from WT, C3KO ND and C3KO BTZ diaphragm muscle samples. Scale bars: 100 μm for lower magnification images, and 50 μm for insets. **(C)** Ubiquitin proteasome activity measured by fluorometric enzymatic assay in muscles from control, non-treated C3KO and BTZ-treated C3KO mice. Data represented as mean % over control ± SEM. BTZ positive control levels are shown as a discontinuous line. *n* = 6 control; *n* = 3 C3KO ND; *n* = 3 BTZ-treated C3KO mice (Kruskal-Wallis post hoc Dunn’s multiple comparisons test). **(D)** Western blot analysis of Ca^2+^-handling proteins in diaphragms from Control, C3KO and BTZ-treated (0.8 mg/kg) mice. Protein signals were normalized to total protein (gel load). **(E)** Quantification of SERCA1, SERCA2, and p-CaMKII protein levels normalized to total protein. Data expressed as mean fold-change ± SEM. *n* = 5–9 per group (Kruskal-Wallis post hoc Dunn’s multiple comparisons test).

After verifying that SERCA protein levels were reduced in the CAPN3-deficient C3KO mouse model, we decided to analyze the effect of UPS inhibition on SERCA proteins. To be able to evaluate the effect of BTZ on the dystrophic phenotype, we selected 9-month-old C3KO mice, in which the dystrophic phenotype is reportedly more evident. Based on a previous study in the Duchenne animal model mdx mice (Gazzerro et al., 2010), 0.8 mg/kg BTZ (Velcade^®^) was administered twice weekly intravenously for 3 weeks. As to the evaluation of body weight in control, C3KO, and treated-C3KO mice, no significant results were obtained between C3KO groups when Two-way ANOVA post hoc Tukey’s multiple comparisons test was applied. (Figure 4A). Furthermore, hematoxylin and eosin (H&E) staining images showed that the treatment did not affect the overall morphology of the muscle (Figure 4B). Next, we assessed the activity of the UPS degradation pathway in the muscles of non-treated (WT and C3KO) and BTZ treated mice (C3KO+BTZ). Surprisingly, we found no differences in the UPS activity levels among the three groups. This result suggests an inefficient inhibition of UPS with the BTZ dose administered (Figure 4C). *Ex vivo* addition of 1uM BTZ to a non-treated WT sample (BTZ positive control) resulted in a 30% decrease in UPS activity (dashed line), which supports the methodological soundness. After evaluation of protein levels in the diaphragm muscles of these animals (Figures 4D,E), we did not find any rescue of SERCA proteins after treatment with BTZ. These results suggest that in our mouse model the administered doses of BTZ did not effectively inhibit UPS and, consequently, higher doses will be needed to test whether SERCA proteins could be rescued *in vivo* with UPS inhibitors in this model.

## Discussion

The present study aimed to evaluate the therapeutic potential of BTZ, a specific UPS inhibitor, in cellular and animal models of LGMDR1 muscular dystrophy. We found that BTZ (5 nM, 24 h) was able to partially rescue diminished SERCA2 protein levels observed in CAPN3-knockdown human myotubes. Moreover, BTZ was also able to restore the basal intracellular Ca^2+^ levels compared to non-treated CAPN3-deficient 8,220 myotubes, which presented a 27% increase in basal intracellular [Ca^2+^]. This result is consistent with our previous studies in another human myogenic line knockdown for CAPN3 (Toral-Ojeda et al., 2016; Toral-Ojeda et al., 2018).

In contrast to the CAPN3 knockdown model, the use of immortalized myoblasts from LGMDR1 dystrophic patients allows for analysis of the effect of specific mutations on pathological features (Mamchaoui et al., 2011). This is particularly relevant for CAPN3, which has a dual catalytic and structural function (Ojima et al., 2011), and where not all mutations affect CAPN3 expression or function equally. Indeed, *CAPN3* mutations do not always result in reduced protein levels (Sáenz et al., 2005). In this study, we have characterized myotubes from two LGMDR1 patients carrying different missense mutations. First, KM900 (LG1) carries a homozygous c.1699G>T mutation first described in 1997 (Richard et al., 1997), for which we found no published information on CAPN3 expression or function. Second, the 918 (LG2) cell line carries c.1637G>T and c.865C>T mutations. c.865C>T mutation has been previously described in combination with c.550delA mutation to severely reduce CAPN3 expression and suppress its catalytic activity (Milic et al., 2007).

Here, we describe that CAPN3 protein is present in LG1 myotubes, but virtually absent in LG2 myotubes. The absence of CAPN3 in LG2 myotubes seems to be accompanied by a marked reduction in SERCA1 and SERCA2 protein levels. These data suggest that the non-proteolytic function of CAPN3 plays an essential role in maintaining proper SERCA protein expression. Noteworthy, in general, patients carrying two null mutations develop a more severe phenotype with an earlier onset, compared to patients carrying at least one missense mutation (Fardeau, et al., 1996a; Fardeau, et al., 1996b; Chae et al., 2001; Paula et al., 2002; Sáenz et al., 2005; Fanin et al., 2007). Thus, if mutant CAPN3 preserves its structural function, SERCA protein would be partially retained, and Ca^2+^ homeostasis would not be as affected, resulting in an overall milder dystrophic phenotype. In this line, studies performed in LGMDR1 mouse models with inactive *Capn3* expression or *Capn3* knockout mice demonstrate that calcium homeostasis is predominantly altered in the absence of CAPN3 (Ojima et al., 2011).

**FIGURE 5:**
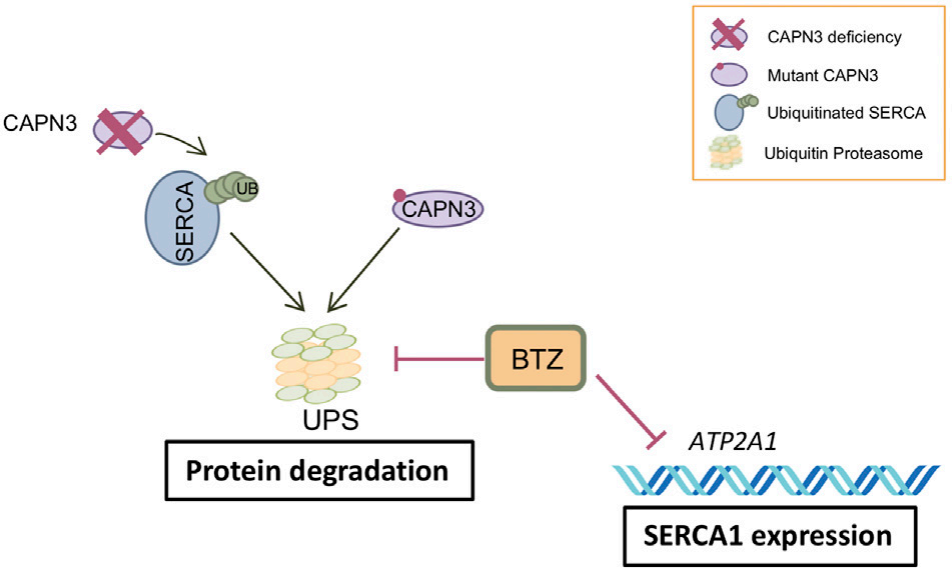
Model of BTZ effect on CAPN3-deficient muscle fibers. CAPN3 deficiency induces increased ubiquitination and degradation of SERCA through the ubiquitin proteasome pathway (UPS). Bortezomib inhibits proteasome activity and thus degradation of SERCA proteins. However, BTZ also strongly inhibits SERCA1 expression at the mRNA level, so that BTZ rescue is mostly observed on SERCA2 proteins. On the other hand, BTZ is able to rescue mutant CAPN3 protein levels carrying missense mutations.

Treatment of human myotubes from LGMDR1 patients with BTZ constitutes a proof of concept for the potential efficacy of UPS inhibition in rescuing expression of mutated CAPN3 proteins as well as SERCA2 proteins in LGMDR1 (Figure 5). However, a major concern is the downregulation of SERCA1 observed at the mRNA level in CAPN3 knockdown myotubes and in myotubes from LGMDR1 patients. Further studies are needed to elucidate whether this effect is related to *in vitro* models or if it also occurs *in vivo*. The latter would likely limit the value of BTZ as a therapeutic strategy against muscular dystrophies. BTZ reversibly inhibits the chymotrypsin-like activity at the β5-subunit of the 20S proteasome core (PSMB_5_) and the trypsin-like activity at the β1-subunit, to a lesser extent (Lü and Wang 2013). Thus, an exciting possibility would be to target other components of the proteasome, such as the muscle E3 ubiquitin ligase MURF1, which is involved in muscle atrophy and has been found upregulated in LGMDR1 muscle biopsies in a previous study (Fanin et al., 2013).

Up to date, different LGMDR1 mouse models have been generated, being the knockout models for *Capn3* the most widely used. Most of the tests carried out for the functional characterization of the C3KO mice have been developed in adult animals. Interestingly, it has been recently shown that calcium dyshomeostasis may be an early phenomenon in this model (DiFranco et al., 2016). In contrast to reports from C3KO mice beyond 4 months old (Ermolova et al., 2011; Kramerova et al., 2016; Liu et al., 2020), we found that 2-month-old C3KO mice show similar performance in grip strength and resistance to fatigue tests compared to controls, suggesting that at this age C3KO are presymptomatic. Additionally, hyper-CKemia has been previously described in the early stages of the disease (Urtasun et al., 1998; Fanin and Angelini 2015). Creatine kinase (CK) constitutes the most sensitive indicator of muscle damage, and it is the most used enzyme in the diagnosis and monitoring of muscle diseases (Bohlmeyer et al., 1994). However, in our younger C3KO mice no significant differences have been found compared to controls, further supporting that muscle damage is not evident in this presymptomatic stage. Lastly, previous findings have shown reduced SR calcium release in isolated muscle fibers from 4-6-month-old C3KO (Kramerova et al., 2008), while in younger C3KO mice, we have not found any alterations in basal calcium compared to wild-type mice. This may be due to the high calcium buffer capacity of mouse muscle fibers or due to a lack of a calcium-related phenotype at this early stage of the disease (Heizmann, Berchtold, and Rowlerson 1982; Vallejo-Illarramendi et al., 2014).

Next, we analyzed SERCA expression in young C3KO mice to address whether SERCA deficiency is an early feature in LGMDR1 pathology. We decided to focus our analysis on the soleus and diaphragm muscles since they are the most affected muscles in this model (Kramerova et al., 2004). These muscles are comprised of a significant proportion of slow fibers and bear a closer resemblance to human skeletal muscles (Kho et al., 2006). Of note, the diaphragm muscle is considered a fast muscle in the mouse, with 10% type I, 40% type IIA, and 40% type IIX. In contrast, the soleus muscle is considered a mixed fast and slow muscle, with 40% type I, 40% type IIA, and 10% type IIX fibers (Barclay and Weber 2004; Talmadge et al., 2004; Feng et al., 2011). Interestingly, in the soleus muscle previous reports suggest a more significant decrease in the cross-sectional area of fast fibers (28%) compared to slow fibers (21%) in C3KO mice (Kramerova et al., 2004; Ermolova et al., 2011). SERCA1 is mainly expressed in fast type II fibers, and accordingly, SERCA1 but not SERCA2 is significantly downregulated in soleus. In contrast, in the diaphragm, we observe a predominant decrease in SERCA2 protein, which suggest a preferential impairment of slow type I fibers in this muscle. Together, these data suggest that *Capn3* deficiency affects preferentially SERCA1 or SERCA2 depending on the type of muscle.

BTZ is an FDA-approved drug for the treatment of multiple myeloma (Kane et al., 2003). BTZ has also been tested in several animal models of muscular dystrophy, including dystrophin deficiency (Duchenne, Becker) and laminin α2 deficiency (MDC1A and LGMDR23), with data showing promising therapeutic potential (Araújo et al., 2009; Gazzerro et al., 2010; Carmignac et al., 2011; Körner et al., 2014). C3KO mice tolerated BTZ administration for 3 weeks in a similar dosage as previously described (Gazzerro et al., 2010), with no obvious changes in body weight compared to no treated animals. Also, histopathological analysis of the diaphragm showed no overt changes in the diaphragm muscle of treated C3KO mice compared to non-treated. However, we found that BTZ treatment did not inhibit proteasome activity in muscle as expected, which may be explained by variable bioavailability between different mouse strains. In this line, BTZ treatment did not rescue SERCA protein levels in C3KO mice, but given the inefficient UPS inhibition achieved in muscle, we could not conclude a lack of BTZ activity in this regard. In order to address this point, further experiments are needed to determine the appropriate dose of BTZ in the C3KO mouse line. Considering the narrow therapeutic index of BTZ, other therapeutic strategies should also be considered, including SERCA overexpression by AAV-mediated gene therapy (Zsebo et al., 2014; Mázala et al., 2015) or several drugs known to increase SERCA expression or function such as adrenoceptor blockers, adrenergic agonists, hormones, glucocorticoids, natural antioxidants, and small molecule SERCA activators (CDN1163) (Taguchi et al., 2007; Vallejo-Illarramendi et al., 2014; Kang et al., 2016).

In conclusion, we have demonstrated reduced SERCA expression in several cellular and animal models of LGMDR1, which likely constitutes an early preclinical feature in CAPN3-deficient mice. Our results support the involvement of SERCA in the etiopathology of LGMDR1 and lay the foundation to explore SERCA2 as well as UPS as novel molecular targets for the treatment of LGMDR1 muscular dystrophy. Furthermore, our results suggest that UPS is involved in the degradation of mutant proteins with missense mutations. Thus, patients carrying CAPN3 missense mutations may further benefit from proteasome inhibition through the rescue of CAPN3 protein levels. Moreover, this mechanism could also be extended to a wide variety of genetic diseases. Further studies in suitable models will be necessary to demonstrate the therapeutic efficacy of proteasome inhibition for different genetic mutations.

## Data Availability

The raw data supporting the conclusions of this article will be made available by the authors, without undue reservation.
